# Selenite Reduction by *Proteus* sp. YS02: New Insights Revealed by Comparative Transcriptomics and Antibacterial Effectiveness of the Biogenic Se^0^ Nanoparticles

**DOI:** 10.3389/fmicb.2022.845321

**Published:** 2022-03-10

**Authors:** Yuting Wang, Qing Ye, Yujun Sun, Yulu Jiang, Bo Meng, Jun Du, Jingjing Chen, Anna V. Tugarova, Alexander A. Kamnev, Shengwei Huang

**Affiliations:** ^1^Department of Pathology, The First Affiliated Hospital of USTC, Division of Life Sciences and Medicine, University of Science and Technology of China, Hefei, China; ^2^Intelligent Pathology Institute, Division of Life Sciences and Medicine, University of Science and Technology of China, Hefei, China; ^3^Institute of Biomedical and Health Science, School of Life and Health Science, Anhui Science and Technology University, Fengyang, China; ^4^Laboratory of Biochemistry, Institute of Biochemistry and Physiology of Plants and Microorganisms—Subdivision of the Federal State Budgetary Research Institution Saratov Federal Scientific Centre of the Russian Academy of Sciences, Saratov, Russia

**Keywords:** *Proteus* sp. YS02, selenite biotransformation, biogenic selenium nanoparticles, transcriptome, antibacterial effectiveness

## Abstract

Biotransformation of selenite by microorganisms is an effective detoxification (in cases of dissimilatory reduction, e.g., to Se^0^) and assimilation process (when Se is assimilated by cells). However, the current knowledge of the molecular mechanism of selenite reduction remains limited. In this study, a selenite-resistant bacterium was isolated and identified as *Proteus* sp. YS02. Strain YS02 reduced 93.2% of 5.0 mM selenite to selenium nanoparticles (SeNPs) within 24 h, and the produced SeNPs were spherical and localized intracellularly or extracellularly, with an average dimension of 140 ± 43 nm. The morphology and composition of the isolated and purified SeNPs were characterized using dynamic light scattering (DLS), scanning electron microscopy (SEM) with energy-dispersive X-ray (EDX) spectrometry, and Fourier transform infrared (FTIR) spectroscopy. FTIR spectroscopy indicated the presence of proteins, polysaccharides, and lipids on the surface of the isolated SeNPs. Furthermore, the SeNPs showed excellent antimicrobial activity against several Gram-positive and Gram-negative pathogenic bacteria. Comparative transcriptome analysis was performed to elucidate the selenite reduction mechanism and biosynthesis of SeNPs. It is revealed that 197 genes were significantly upregulated, and 276 genes were significantly downregulated under selenite treatment. Gene ontology and Kyoto Encyclopedia of Genes and Genomes (KEGG) analyses revealed that genes associated with ABC transporters, sulfur metabolism, pentose phosphate pathway (PPP), and pyruvate dehydrogenase were significantly enhanced, indicating selenite is reduced by sulfite reductase with PPP and pyruvate dehydrogenase supplying reducing equivalents and energy. This work suggests numerous genes are involved in the response to selenite stress, providing new insights into the molecular mechanisms of selenite bioreduction with the formation of SeNPs.

## Introduction

Selenium (Se) is a metalloid that plays a vital role in maintaining human health (Chen et al., 2021). It can replace sulfur in cysteine and be co-translationally incorporated as selenocysteine (SeCys) in selenoproteins involved in biological processes, including detoxification, anti-inflammation, antioxidant defense, and thyroid functioning. A trace amount of selenium is important for human health. Selenium deficiency in humans has been associated with cardiovascular disease, thyroid dysfunction, and immune system dysfunction (Pedrero et al., 2006). Selenium supplementation can protect animals and ameliorate the toxic effects of heavy metals (Ge et al., 2021), improve the clinical symptoms of heart failure (Al-Mubarak et al., 2021), stimulate the immune response toward cancer cells (Yazdi et al., 2012), and alleviate oxidative stress-induced intestinal epithelial barrier injury (Song et al., 2017a). However, selenium is also known as a “double-edged sword” element—it is essential to human and animal health in trace amounts but is toxic in excess (Nancharaiah and Lens, 2015b). Moreover, the toxicity of selenium is not merely defined by its concentration but is also dependent on its speciation. In nature, selenium exists in different forms, including selenite (SeO_3_^2−^), selenate (SeO_4_^2−^), selenide (Se^2−^), and as an elementary substance (Se^0^). Among these, selenite is the most toxic form for aquatic life and humans because of its high mobility in aqueous environments and high bioavailability. However, elemental selenium is biologically inert and generally thought to be non-toxic (Song et al., 2017a). Furthermore, compared with selenium, nanosized elemental selenium particles possess much higher bioactivity and biosafety properties, including improved antioxidant, anticancer functions, and antibacterial activity. Therefore, selenium nanoparticles (SeNPs) may be a more valuable selenium-adding form and therapeutic agent for human health and are attracting increasing attention.

Microorganisms are crucial in the global biogeochemical cycle of selenium on the Earth’s surface. Microbial transformation of selenite and selenate can occur both aerobically or anaerobically. More importantly, selenite/selenate transformation is often accompanied by the formation of SeNPs existing either inside the cytoplasm, within the periplasm, or extracellularly (Ojeda et al., 2020). Se^0^ particles formed by Se-oxyanion reducing microorganisms, including *Azospirillum thiophilum* (Tugarova et al., 2018), *Azospirillum brasilense* (Kamnev et al., 2017, 2021), yeast (Kieliszek et al., 2015, 2016; Jiménez-Lamana et al., 2018), *Enterobacter cloacae* Z0206 (Song et al., 2017a), *Stenotrophomonas maltophilia* SeITE02 (Lampis et al., 2017), *Bacillus* sp. Y3 (Yasir et al., 2020), *Comamonas testosteroni* S44 (Tan et al., 2018), etc., have been reported. So far, it has been found that bacterial Se(IV) reduction may occur through enzymatic or nonenzymatic mechanisms (Tugarova and Kamnev, 2017). Specifically, the non-enzymatic reduction of selenite is mediated by biogenic glutathione, iron siderophores, and sulfide (Nancharaiah and Lens, 2015a). For enzyme-dependent reduction, reductases encompassing sulfite reductase (Huang et al., 2021), glutathione reductase (Wang et al., 2019), thioredoxin reductase (Hunter, 2014), SerT (Tan et al., 2018), flavoprotein CsrF (Xia et al., 2018), and fumarate reductase (Song et al., 2017b) have been reported to be potentially involved in Se(IV) reduction in various bacterial species, suggesting the reduction of selenite occurs through diverse mechanisms. However, the current knowledge of the molecular mechanism of microbial selenite reduction is still limited. For example, it is largely unknown how bacterial cells respond to selenite stress and what molecular mechanism is utilized by microorganisms to reduce selenite. Therefore, the comprehensive studies of the mechanism of selenite reduction at the genome level by applying comparative transcriptomics analysis, microarray analysis, and proteomic analysis are urgently needed.

In the present study, the bacterial strain YS02 exhibiting an efficient selenite transformation ability was isolated from soil. Selenite reduction assay showed that strain YS02 can transform 93.2% of 5.0 mM selenite to SeNPs within 24 h, serving as an eco-friendly cell factory for the biogenesis of SeNPs. However, the exact molecular mechanism of selenite reduction by this strain is not clear. Therefore, the transcriptome response of YS02 cells exposed to sodium selenite (Na_2_SeO_3_) was investigated intensively using comparative transcriptomics analysis to clarify the possible mechanism of selenite reduction and biogenesis of SeNPs by the isolate YS02. Furthermore, the antibacterial activity of the SeNPs against Gram-negative and Gram-positive bacteria, such as *Escherichia coli* and *Bacillus subtilis*, was also investigated to explore the potential of the SeNPs as key assets in the future of healthcare.

## Materials and Methods

### Reagents and Medium

Sodium selenite (Na_2_SeO_3_) was obtained from Sigma-Aldrich (St. Louis, MO, United States). YEP (yeast extract peptone) broth was provided by Shanghai Gu Duo Biotechnology Co., Ltd. (Shanghai, China). All other analytical grade reagents used in present study were obtained from Sinopharm Chemical Reagent Co., Ltd. (Shanghai, China) and Solarbio Science and Technology Co., Ltd. (Beijing, China).

### Isolation and Identification of Selenite-Resistant Strains

Soil samples were taken from the seleniferous soil of Shitai county, ChiZhou, Anhui Province, China. The primary screening for microorganisms capable of reducing selenite to elemental Se was performed as described by Wang et al. (2018b) with small modifications. Briefly, 0.1 g of soil sample was suspended in sterile water (1 ml) with continuous shaking at 180 rpm at 30°C for 0.5 h. Then, 100 μl of the soil sample was serially diluted (10-fold) and placed on YEP plates supplemented with 10 mM Na_2_SeO_3_. After incubating at 30°C for 48 h, the colonies with a red color were selected and streak-cultured on new plates until pure cultures were obtained. Among the bacterial isolates, strain 02 (named YS02) was finally selected considering the most promising growth performance and selenite reduction activity.

Genomic DNA of strain YS02 was isolated using a Dzup Genomic DNA Isolation Kit (Sangon Biotech Co., Ltd., Shanghai, China) for the identification of isolate YS02. The 16S rRNA gene fragment amplification and sequencing were conducted as described by Huang et al. (2021). The obtained 16S rRNA gene sequence was then compared with sequences available on EzBioCloud server (Yoon et al., 2017) and a phylogenetic tree based on maximum likelihood was constructed using the MEGA 7.0 software (Kumar et al., 2016). The obtained 16S rRNA gene sequence was submitted to the GenBank database and has been assigned accession number MZ182304.

### Selenite Reduction and Production of SeNPs

The determination of SeO_3_^2−^ reduction efficiency and the amount of Se^0^ produced by isolate YS02 were performed following a previously established protocol (Wang et al., 2018b). Briefly, isolate YS02 was cultured at 30°C in YEP medium containing 5.0 mM selenite. Then, 10 ml of bacterial culture was collected every 3 h. The bacterial growth of the strain was calculated using the plate-counting method. For the selenite reduction assay and calculation of the amount of selenium (Se^0^) formed, samples were centrifuged at 12,000 *g* for 20 min. The remaining levels of Se(IV) were determined using ICP-OES (inductively coupled plasma optical emission spectrometry, Thermo Fisher Scientific, Waltham, MA, United States; Nawaz et al., 2015). At the same time, spectrophotometry was applied to measure the Se^0^ (Khoei et al., 2017) in the pellet obtained after centrifugation.

### Localization of SeNPs

Isolate YS02 was cultured in YEP medium containing 5.0 mM Na_2_SeO_3_ (180 rpm, 30°C). Bacterial cells cultured in YEP medium without Na_2_SeO_3_ were set as a control. After incubation overnight, the samples were collected using gentle centrifugation (5,000 *g*, 5 min).

To use transmission electron microscopy (TEM), the pellets were fixed with glutaraldehyde (2% final concentration), placed onto carbon-coated copper grids and observed at 80.0 kV on a transmission electron microscope (Hitachi HT-7700, Tokyo, Japan). For scanning electron microscopy (SEM), the pellets were fixed overnight at 4°C with glutaraldehyde (2.5% final concentration), followed by dehydration with a 30%, 50%, 70%, 80%, 95%, 100% ethanol gradient. Finally, the samples were processed for critical point drying and observed with a Hitachi S4800 SEM (Tokyo, Japan).

### SeNPs Preparation and Characterization

The preparation and purification of SeNPs from bacterial cultures of YS02 were conducted using a previously published protocol of Wang et al. (2018b). The particle size and zeta-potential of the obtained SeNPs were measured by dynamic light scattering (DLS; Zen 3600 Zetasizer Nano-ZS, Malvern Instruments Ltd., Worcestershire, United Kingdom; Lampis et al., 2017). The chemical composition of the SeNPs was determined by energy-dispersive X-ray (EDX), while the morphology of the SeNPs was observed by SEM.

For FTIR spectroscopic analysis, SeNPs were separated from cells and cell debris in the bacterial culture after incubation with sodium selenite by centrifugation at 6,000 *g* for 5 min. After centrifugation, the supernatant was collected and filtered through a 0.22 μm filter. Then, SeNPs were harvested by centrifuging the resulting filtered supernatant (40,000 *g*; 30 min); the obtained pellet was washed three times with ddH_2_O and finally resuspended in ddH_2_O; the suspension was dried in a vacuum freeze-drying system. A mid-infrared spectrum (4,000–400 cm^−1^) of the freeze-dried SeNPs (in a KBr pellet, using KBr heated at 150°C for 5 min prior to its use for pelleting, prepared at ambient conditions under a pressure of 20 MPa for 40 s) was recorded in the transmission mode using a Thermo Scientific Nicolet iS20 FTIR spectrometer (Waltham, MA, United States). The baseline-corrected spectroscopic data were collected and manipulated using the OMNIC software (ver. 8.2.0.387); no automatic smoothening was applied, as the spectrum was of appropriate quality.

### Antibacterial Activity of SeNPs

The antibacterial activity of SeNPs produced by strain YS02 was measured determined using the cup diffusion method as described by Al Jahdaly et al. (2021). To prepare water-dispersed SeNPs, 20 mg of SeNPs were suspended in sterile ddH_2_O to obtain a concentration of 200 mg/ml. Two Gram-positive bacteria including *Staphylococcus epidermidis* (ATCC-51625), and *B. subtilis* (ATCC-6633), and two Gram-negative bacteria including *Pseudomonas aeruginosa* (ATCC-47085), and *E. coli* (ATCC-8739), were used in this study. Briefly, the four bacteria were cultured in a shaking incubator (180 rpm) at 30°C (*P. aeruginosa*) or 37°C (other organisms) for 24 h, and then seeded in Petri dishes containing agar media. Then a droplet of 50 μl of the SeNPs suspension or a Kanamycin solution (100 μg/ml) was added onto filter paper disks (ø 6 mm) and left to dry. Finally, the disks with the investigated SeNPs and with the standard antibiotic were placed on the agar plates and incubated at 30°C (*P. aeruginosa*) or 37°C (other organisms). After 24 h of incubation, the antibacterial activity was evaluated by measuring the size of the inhibition zone.

### Transcriptomic Analysis

#### Total RNA Extraction, Library Preparation, and Sequencing

Isolate YS02 was cultured under the same culture conditions in YEP medium containing 5.0 mM selenite (selenite treatment) or without selenite (control). After batch culturing at 30°C for 14 h, YS02 cells were harvested, quenched in liquid nitrogen immediately for 5 min, and stored at −80°C until use. The samples from control were defined as CK-1, CK-2, and CK-3, while samples from selenite treatment were set as Se-1, Se-2, and Se-3, respectively. The total RNA extraction, RNA integrity assessment, and cDNA library construction were conducted at Beijing Genomics Institute (BGI, Shenzhen, China) according to the methods described by Wang et al. (2021). Finally, RNA-*sequencing* of the cDNA library was carried out on a BGISEQ-500 platform (BGI, Shenzhen, China). After quality control and removing adaptor sequences/low-quality sequences (Sun et al., 2019), the clean reads that were filtered from the raw reads were mapped to the reference genome sequences of *P. mirabilis* ATCC 29906 (genome assembly ASM16075v1) using HISAT (Hierarchical Indexing for Spliced Alignment of Transcripts). The raw data produced in the present study have been deposited in the National Center for Biotechnology Information (NCBI) database under the accession number PRJNA737579.

#### Transcriptomic Analyses

The expression levels of different genes and transcripts were measured using FPKM (fragments per kilobase per million mapped fragments). Differential expression analysis between the two groups (CK vs. selenite treatment) was performed using the DESeq2 Bioconductor package. Gene abundance ratio log_2_ (fold change; log_2_(FC)) ≥ 1 or ≤ −1 and False Discovery Rate (FDR) < 0.05 were defined to be regulated differently. GO-Term Finder was used to identify gene ontology (GO) terms, and GO enrichment analysis was performed to evaluate significantly over-represented functional categories using Fisher’s exact test with an FDR threshold of 5%. Kyoto Encyclopedia of Genes and Genomes (KEGG) enrichment analysis was conducted to understand high-level functions and utilities of differentially expressed genes (DEGs; Yu et al., 2019).

#### Quantitative Real-Time PCR Validation

To verify the reliability of the transcriptome sequencing data, several representative DEGs were selected for quantitative analysis. These genes encode key enzymes involved in the glutathione and sulfur metabolism, pyruvate dehydrogenase system, and pentose phosphate pathway (PPP). Primer sequences used in this study are listed in Supplementary Table S2. Isolate YS02 was cultured in the condition that mimicked the conditions used for the RNAseq library preparation described above. The total RNA was isolated using a Total RNA Extraction Kit (Solarbio Science and Technology Co., Ltd., Beijing, China). Then PCR reactions were performed using One Step RT-qPCR Kit (Sangon Biotech Co., Ltd., Shanghai, China), in accordance with the manufacturer’s protocol. The 16S rRNA gene was used as a reference gene, and the fold changes in target gene expression was calculated using the 2^−ΔΔCt^ method.

## Results and Discussion

### Bacterial Strain Isolation and Identification

**Figure 1:**
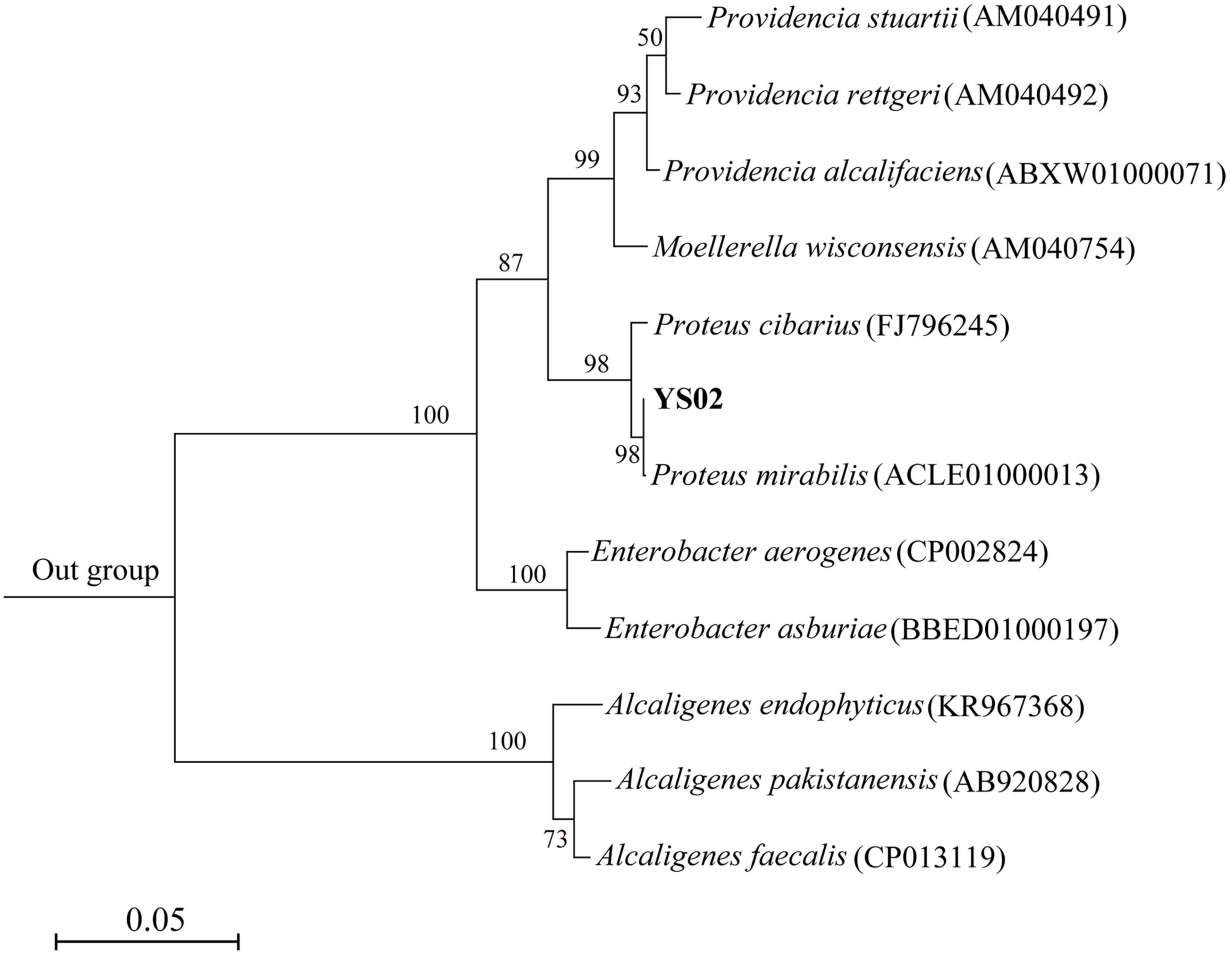
Maximum likelihood tree inferred through MEGA 7 software based on 16S rRNA gene sequence of strain YS02 and related representative strains. *Sphingobacterium zeae* (KU201960) was used as the out-group member. The scale bars represent 0.05 substitutions per site.

Microbial and enzymic activities in soil play a critical role in the global selenium cycle; thus, Se-rich sediments or soils are an ideal source for isolating selenite-reducing bacteria (El-Ramady et al., 2015; Eswayah et al., 2016). In this study, 15 bacterial strains were isolated from seleniferous soil. Of these, isolate 02 (YS02) showed the best growth performance on YEP plates containing 10 mM Na_2_SeO_3_. BLAST search results indicated that the 16S rRNA gene sequence of strain YS02 exhibited 99% sequence similarity to that of *Proteus mirabilis* ATCC 29906(T). Furthermore, phylogenetic analysis also showed that strain YS02 belongs to the genus *Proteus*, with the highest similarity to *P. mirabilis* (Figure 1). Therefore, the strain YS02 was identified as *Proteus* sp. YS02. *Proteus* spp. are widely distributed in the environment, including animal guts, soils, and water. They possess the ability to tolerate or utilize polluting compounds (e.g., heavy metals and antibiotics) and promote plant growth. Moreover, several members of the genus *Proteus*, such as *P. mirabilis* YC801 (Wang et al., 2018a) or *P. hauseri* QW4 (Khalilian et al., 2015), have been reported to reduce selenite/selenate to Se^0^ and biosynthesize SeNPs, allowing for the possibility of exploring these microorganisms in selenium bioremediation and synthesis of SeNPs. Therefore, the isolate YS02 was selected for subsequent study.

### Synthesis of SeNPs by *Proteus* sp. YS02 and Their Characterization

**Figure 2:**
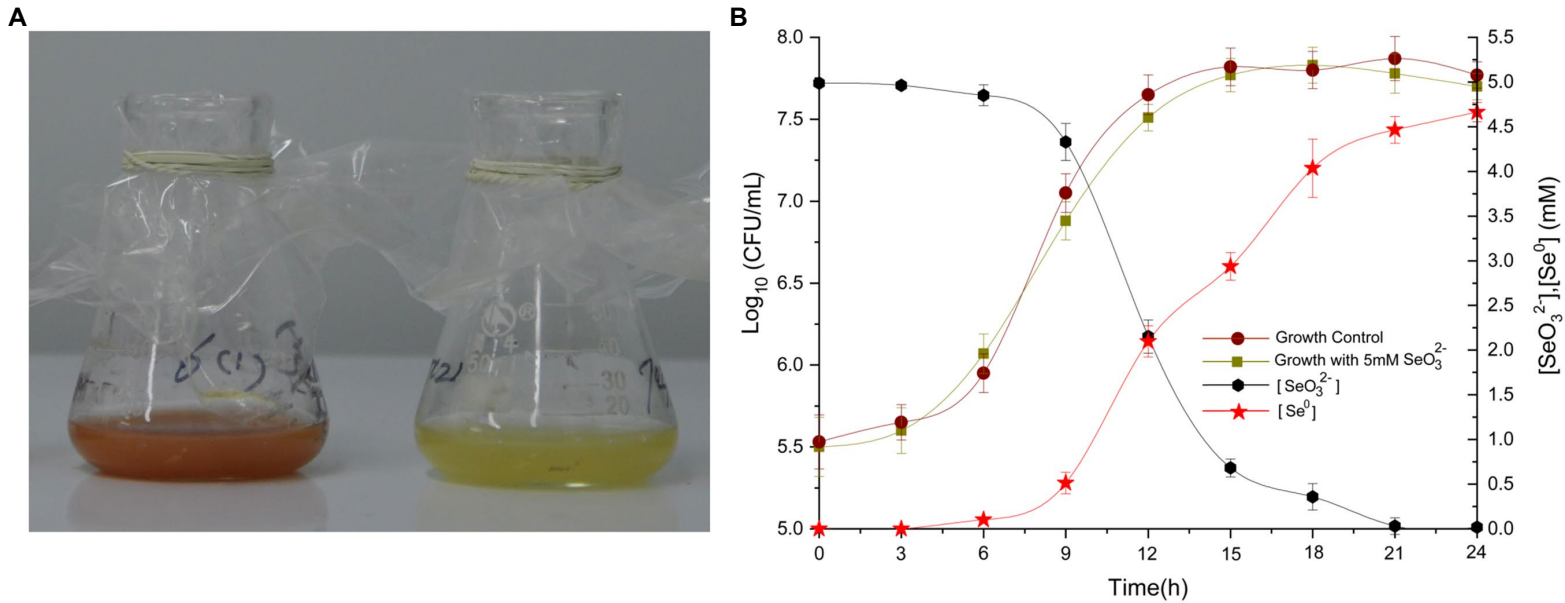
Growth of bacterial strain YS02 in liquid YEP medium containing 5.0 mM selenite. **(A)** Images of cultures with 5.0 mM selenite (on the left) and without selenite (on the right) aerobically grown for 9 h and **(B)** the growth curve, time courses of SeO_3_^2−^ reduction, and Se^0^ production by strain YS02. Each test was performed in triplicate, and data were presented as the mean ± standard deviation.

After cultivation of *Proteus* sp. YS02 in selenite-containing YEP culture medium, SeO_3_^2−^ reduction and Se^0^ formation were observed within 6 h indicated by the formation of a reddish coloration in the culture medium that is a typical characteristic of microbially produced Se^0^ (Figure 2A). Furthermore, it is interesting that the SeO_3_^2−^ reduction and Se^0^ biogenesis were in accordance with the strain growth kinetics; only 2.9% of the initial SeO_3_^2−^ were reduced within 6 h, while most of the remaining selenite (>73%) was depleted during the exponential growth phase (between 9 and 15 h), and then nearly exhausted after 24 h of incubation (Figure 2B). Selenite reduction being tightly related to the bacterial growth phase has been reported for other bacteria, including *Alcaligenes faecalis* Se03 (Wang et al., 2018b), *S. maltophilia* SeITE02 (Lampis et al., 2017), and *Lysinibacillus* sp. (Zhang et al., 2019), suggesting that reducing compounds and/or cellular reductases (whose secretion and consumption are inextricably associated with the growth state of the microbes; Wang et al., 2018b) catalyze selenite reduction. Moreover, the depletion of SeO_3_^2−^ was accompanied by the production of Se^0^—after 24 h of cultivation, about 93.2% of the initial selenite were transformed to Se^0^ (Figure 2B). In contrast, no visible color change or Se^0^ production was noted in control flasks that contained merely strain YS02 (see Figure 2A, right-hand flask) or selenite (data not shown), suggesting the active participation of bacterial isolate YS02 in selenite biotransformation and Se^0^ production.

**Figure 3:**
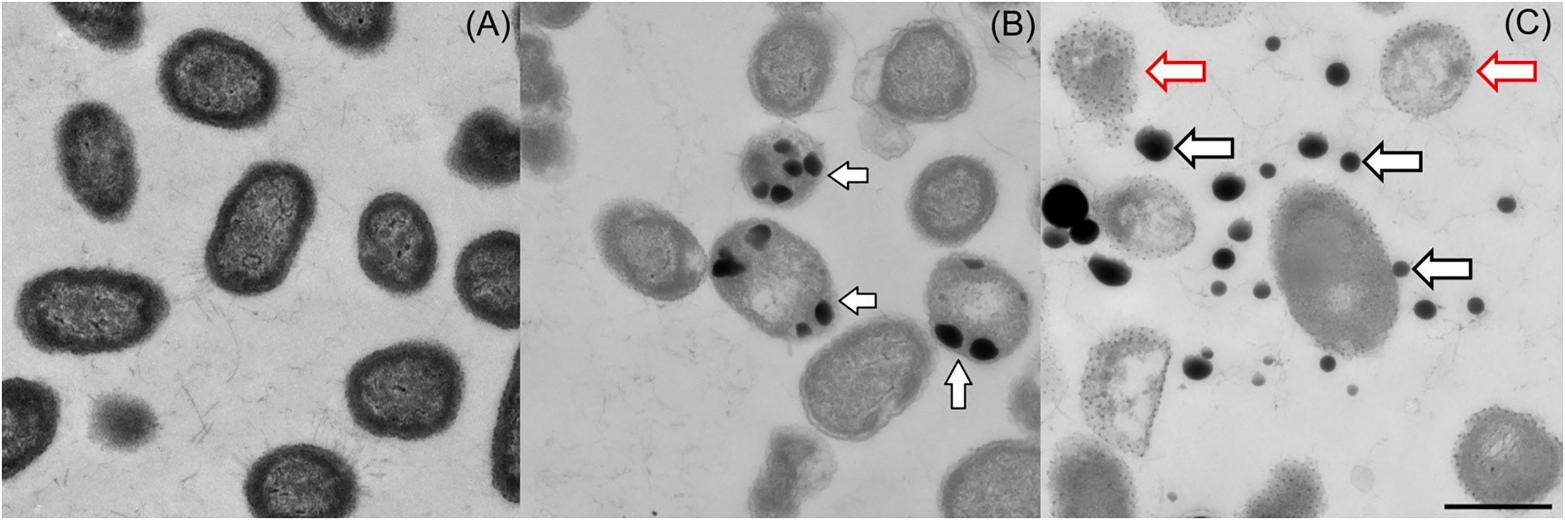
TEM analysis of *Proteus* sp. YS02 **(A)** cultured without Na_2_SeO_3_ and **(B,C)** cultured with 5 mM Na_2_SeO_3_ after 24 h of incubation. White arrows show nanoparticles inside **(B)** or outside **(C)** the cells. Empty ghost cells are indicated by red arrows **(C)**. The bar represents 1 μm.

**Figure 4:**
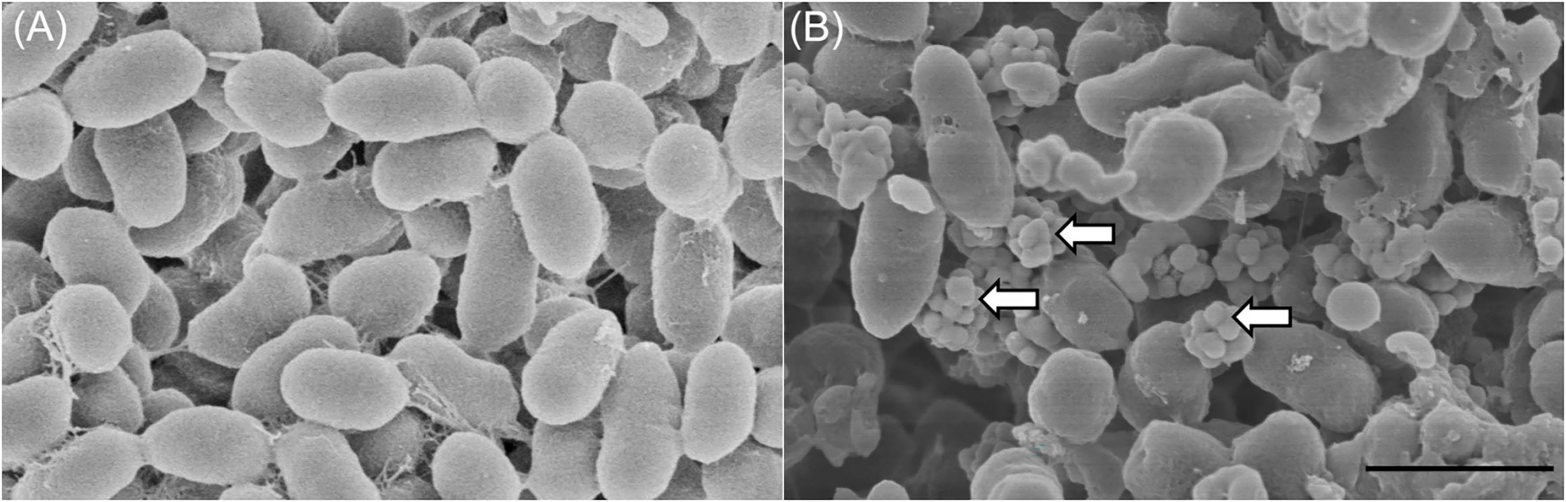
SEM analysis of *Proteus* sp. YS02 **(A)** cultured without Na_2_SeO_3_ and **(B)** cultured with 5 mM Na_2_SeO_3_ for 24 h. White arrows indicate the produced extracellularly located nanoparticles. The bar represents 1 μm.

As many bacteria link the bioreduction of selenite to Se^0^ with the production of SeNPs, we investigated whether *Proteus* sp. YS02 has the capacity to transform selenite to SeNPs. As shown in Figure 3, TEM analysis clearly revealed electron-dense nanoparticles (Se^0^ nanoparticles) both inside and outside of the cells. However, the Se^0^ nanoparticles could be observed mainly in the extracellular space (Figure 3C). Meanwhile, the formation of nanoparticles was not detected in cultures grown in the absence of selenite (Figure 3A). Interestingly, some extracellular nanoparticles appeared to be associated with empty ghost cells (cell walls appearing damaged; indicated by red arrows in Figure 3C), suggesting that this extracellular location is probably the consequence of cell lysis. Note that extracellular Se^0^ nanoparticles have also been reported to be associated with empty ghost cells or cellular debris in other bacteria, such as *Vibrio natriegens* (Fernández-Llamosas et al., 2017), *S. maltophilia* SeITE02 (Lampis et al., 2017), and *Bacillus mycoides* SeITE01 (Lampis et al., 2014), which indicates that cell lysis may cause the release of intracellularly formed Se^0^ nanoparticles in these bacteria. Moreover, SEM analyses (Figures 4A,B) also confirmed the presence of extracellular Se^0^ nanoparticles (Figure 4B, white arrows). The micrographs showed an accumulation of electron-dense particles attached to the outer side of the external cell, while these particles were not observed in the cells grown on YEP without the selenite (see Figure 4A). Moreover, the Se^0^ nanoparticles appeared spherical in shape and decidedly dishomogeneous in terms of size.

**Figure 5:**
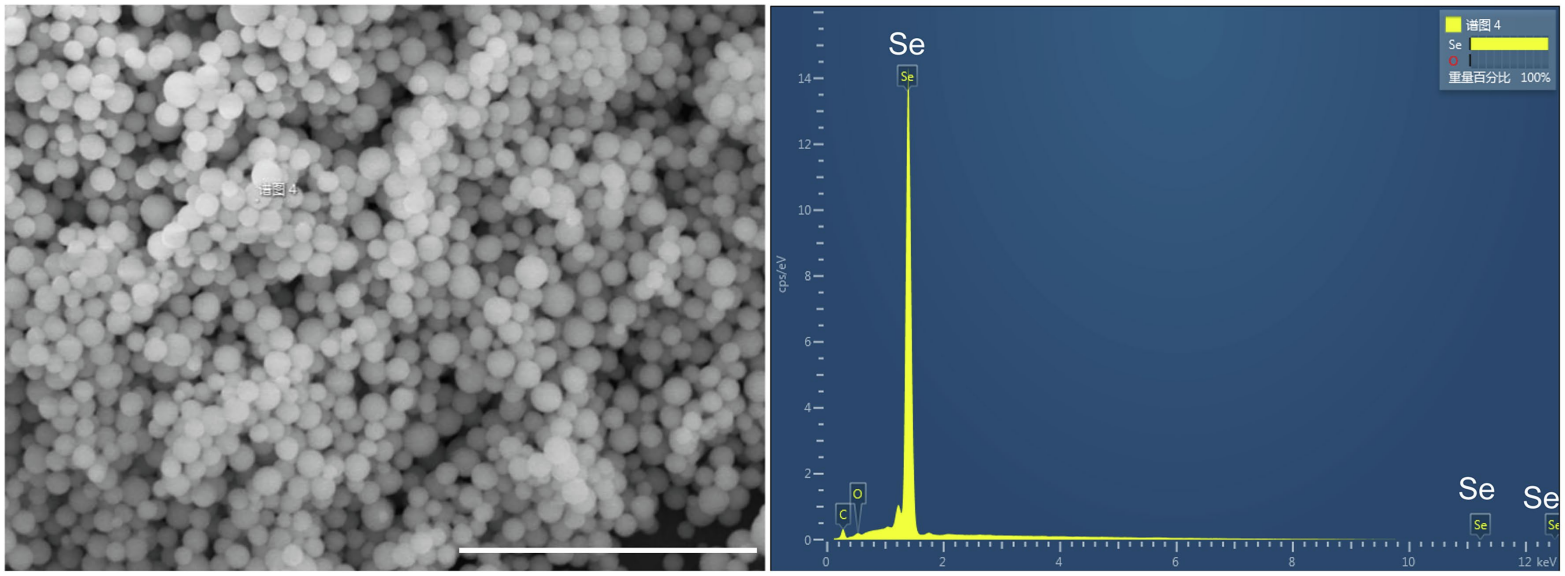
SEM–EDX analysis of purified SeNPs produced by *Proteus* sp. YS02. The bar represents 5 μm.

The produced Se^0^ nanoparticles were purified and further analyzed by SEM–EDX analysis. As shown in Figure 5, the purified SeNPs appear as spherical nanoparticles of varied sizes. The EDX spectra also clearly confirmed the presence of selenium—the spherical nanoparticles exhibit Se-specific absorption peaks at 1.37, 11.22, and 12.49 keV. Moreover, DLS analysis of purified SeNPs revealed an average dimension of 140 ± 43 nm, similar to those found in *Azoarcus* sp. CIB (174 ± 36 nm; Fernández-Llamosas et al., 2016) and in *V. natriegens* (136 ± 31 nm; Fernández-Llamosas et al., 2017). More, the produced SeNPs revealed a negative zeta-potential (−34.2 mV), suggesting the colloidal stability of SeNPs in the water phase.

**Figure 6:**
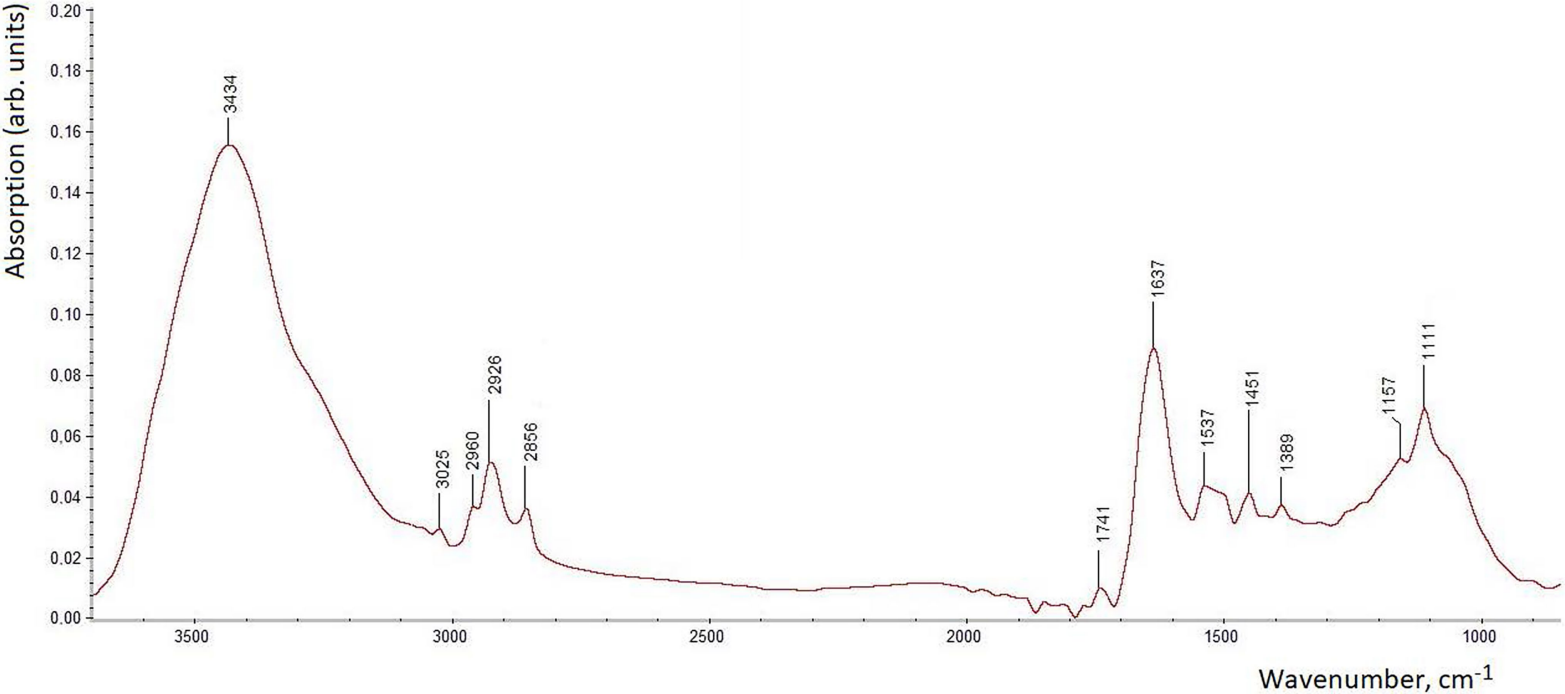
Fourier transform infrared spectrum of isolated SeNPs produced by *Proteus* sp. YS02.

In order to investigate the possible existence of bioorganic capping layers that could contribute to the synthesis and stability of SeNPs, FTIR spectroscopic analysis of the isolated and purified SeNPs was performed (Figure 6). This technique is highly sensitive to the presence of biomaterials covering the SeNPs surface and has been widely used for their characterization (see, e.g., Kamnev et al. (2017, 2021), Tugarova et al. (2018) and references cited therein). The FTIR spectrum in Figure 6 shows a number of absorption bands typically observed for biogenic SeNPs of microbial origin. The main important bands include: a very strong broad non-symmetric envelope around 3,600–3,200 cm^−1^ (featuring stretching vibrations of H-bonded O–H and N–H moieties); a series of bands at ~3,000–2,800 cm^−1^ (various characteristic stretching vibrations of C–H bonds in alkanoic groups); weak but typical bands at 1,741 cm^−1^ (stretching vibrations of the C=O group in ester moieties), 1,451 cm^−1^ (bending vibrations of –CH_3_/–CH_2_– groups) and 1,389 cm^−1^ (symmetric stretching vibrations of carboxylic –COO^−^ groups in amino acid side chains and/or carboxylated polysaccharides); a characteristic pair of bands between 1,637 and 1,537 cm^−1^ (the amide I and II bands of proteins, respectively); a series of overlapping bands at ~1,200–950 cm^−1^ featuring polysaccharides and, occasionally, phosphate moieties (Kamnev et al., 2017, 2021; Tugarova et al., 2018).

Thus, in accordance with the FTIR spectroscopic data, the biomacromolecular composition of the obtained biogenic SeNPs most likely includes proteins (for which the amide I and II bands mentioned above are accompanied by the weaker absorption around 1,240 cm^−1^ featuring another typical protein-related band, amide III; Kamnev et al., 2017, 2021; Tugarova et al., 2018), polysaccharides (see the increased O–H stretching envelope together with the characteristic region at ~1,200–950 cm^−1^) and lipids (featured by a combination of stretching and bending C–H vibrations with the ester carbonyl band at 1,741 cm^−1^). As was mentioned above, these biomacromolecular components are often found in surface capping layers of SeNPs produced by various microorganisms.

Note that carboxylic groups (featured by the weak band at 1,389 cm^−1^ ascribed to their symmetric stretching vibrations; the accompanying antisymmetric vibrations of variable location at higher wavenumbers are known to be often masked by stronger amide I/amide II bands; Kamnev et al., 2017, 2021; Tugarova et al., 2018) are responsible for the negative zeta potentials typically found for such SeNPs in aqueous suspensions (Tugarova and Kamnev, 2017). Finally, traces of water (which can form strong H-bonds with polar biomolecular groups and thus might be not fully removed by freeze-drying; see “SeNPs Preparation and Characterization”) could contribute to the increased stretching O–H region (3,600–3,200 cm^−1^) as well as indirectly to the amide I region by its bending (scissoring) H–O–H vibrations which are observed at ~1,640–1,650 cm^−1^ (Kamnev et al., 2021).

### Antibacterial Activity

So far, a lot of metallic and metalloid nanoparticles have been reported to possess antimicrobial activity and thus can be exploited for alternative medicine therapy against multidrug-resistant pathogenic microbes. To evaluate potential biomedical utilizations of the SeNPs produced by YS02, their antibacterial activity against different specific strains including both Gram-negative (*P. aeruginosa* and *E. coli*) and Gram-positive (*B. subtilis* and *S. epidermidis*) bacteria was evaluated by the plate antibacterial test.

**Figure 7:**
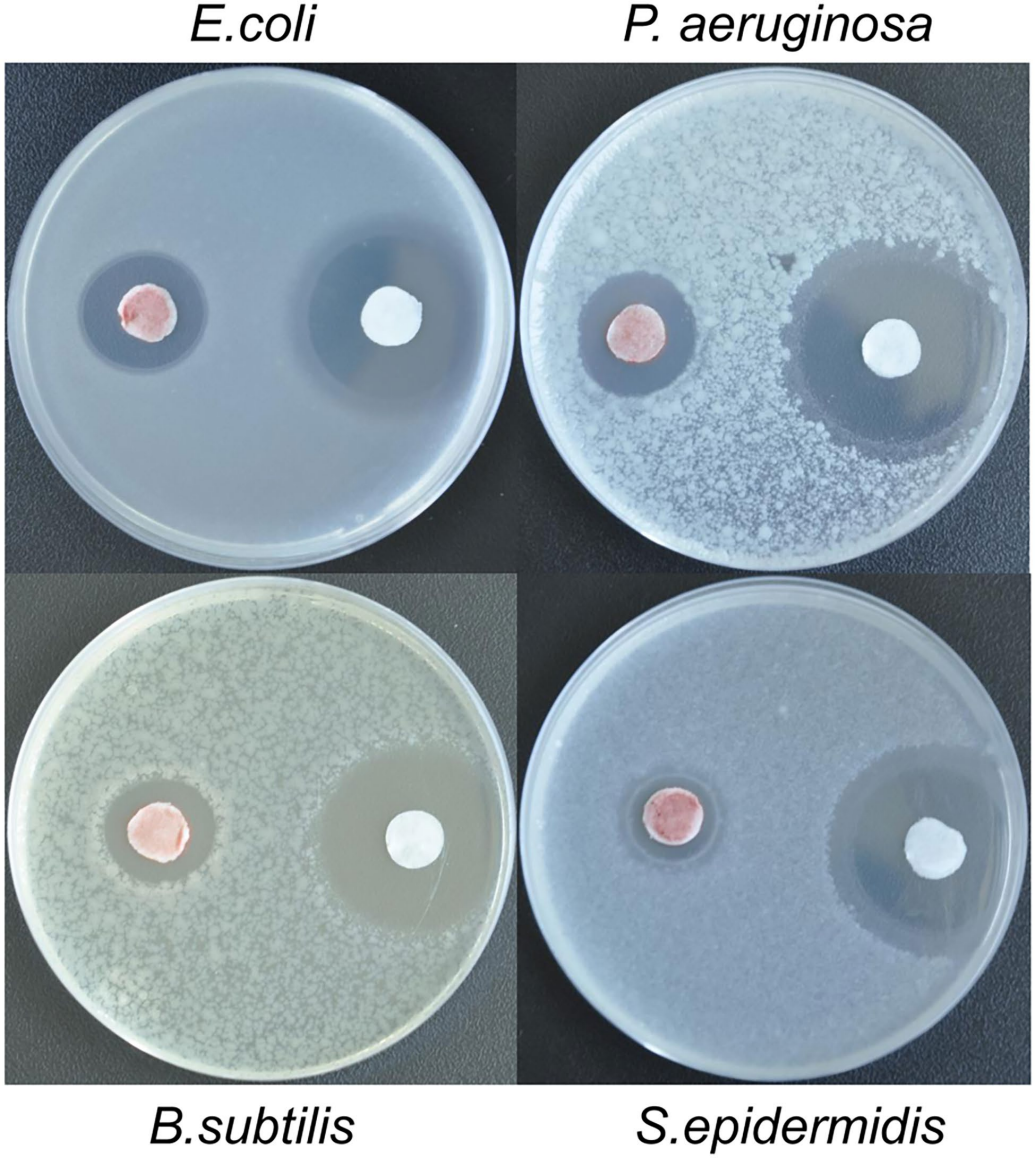
The antibacterial effect of isolated SeNPs (produced by *Proteus* sp. YS02) on *E. coli*, *P. aeruginosa*, *B. subtilis*, and *S. epidermidis* screened by the plate antibacterial test. The red colored disks represented SeNPs and the white disks represented the standard antibiotic.

As shown in Figure 7, SeNPs at a specific concentration could effectively inhibit the microbial growth. Furthermore, it is interesting that the growth of the Gram-negative bacteria (*P. aeruginosa* and *E. coli*) was more influenced by the SeNPs compared to that of the Gram-positive bacteria as indicated by corresponding inhibition zones. Application of antibiotic (Kanamycin) provided the highest growth inhibition zones with sizes of 18.4 mm and 22.9 mm for *E. coli* and *P. aeruginosa*, respectively. However, the SeNPs also showed significant antibacterial effect with inhibition zone sizes between 13.5 and 12.5 mm for *E. coli* and *P. aeruginosa*, respectively. For the Gram-positive bacteria, smaller inhibition zones were observed after the application of SeNPs: the sizes of inhibition zones were 11.9 mm for *B. subtilis* and 9.8 mm for *S. epidermidis*. Similarly, it was also found that the growth of *E. coli* and *P. aeruginosa* was most influenced by PVA/Chitosan/SeNPs nanocomposite while the effect on *B. subtilis* was the lowest (Menazea et al., 2020). These results could be attributed to the distinct differences in the cell wall structure/cell surface between Gram-positive and Gram-negative bacteria—the former have a stronger molecular network in the cell wall, which could present more difficulties for selenite ions to penetrate the cell than in Gram-negative bacteria (Truong et al., 2021). It has recently been reported that the bactericidal activity of SeNPs can be explained in terms of changing the membrane potential, depleting ATP, promoting ROS production, and disrupting the membrane (Huang et al., 2020).

Overall, the findings reported in the present study indicate that the SeNPs produced by YS02 have a strong bactericidal effect on the pathogenic bacteria and have a potential to be used as a substitute for antibiotics in clinical applications. However, further research is needed to address the precise mechanism of the antibacterial action of the SeNPs.

### Overview of *Proteus* sp. YS02 Transcriptomic Profile

Although selenite reduction mechanisms and SeNPs synthesis have been studied in several bacterial species, they have not been completely clarified (Tugarova and Kamnev, 2017; Tugarova et al., 2020). The global transcriptome of the *Proteus* sp. YS02 cells was analyzed by RNA-seq technology to clarify the molecular mechanism of selenite reduction and production of SeNPs. Overall, six libraries (CK-1, CK-2, CK-3, Se-1, Se-2, and Se-3) were generated and sequenced using a DNBSEQ-T7 sequencer, and about 255 million pair-end clean reads (2 × 150 bp long) were obtained. The number and quality scores of the aligned reads are shown in Supplementary Table S1 and were sufficient to ensure the accuracy of the assembly and coverage.

### Transcriptional Data Analysis

**Figure 8:**
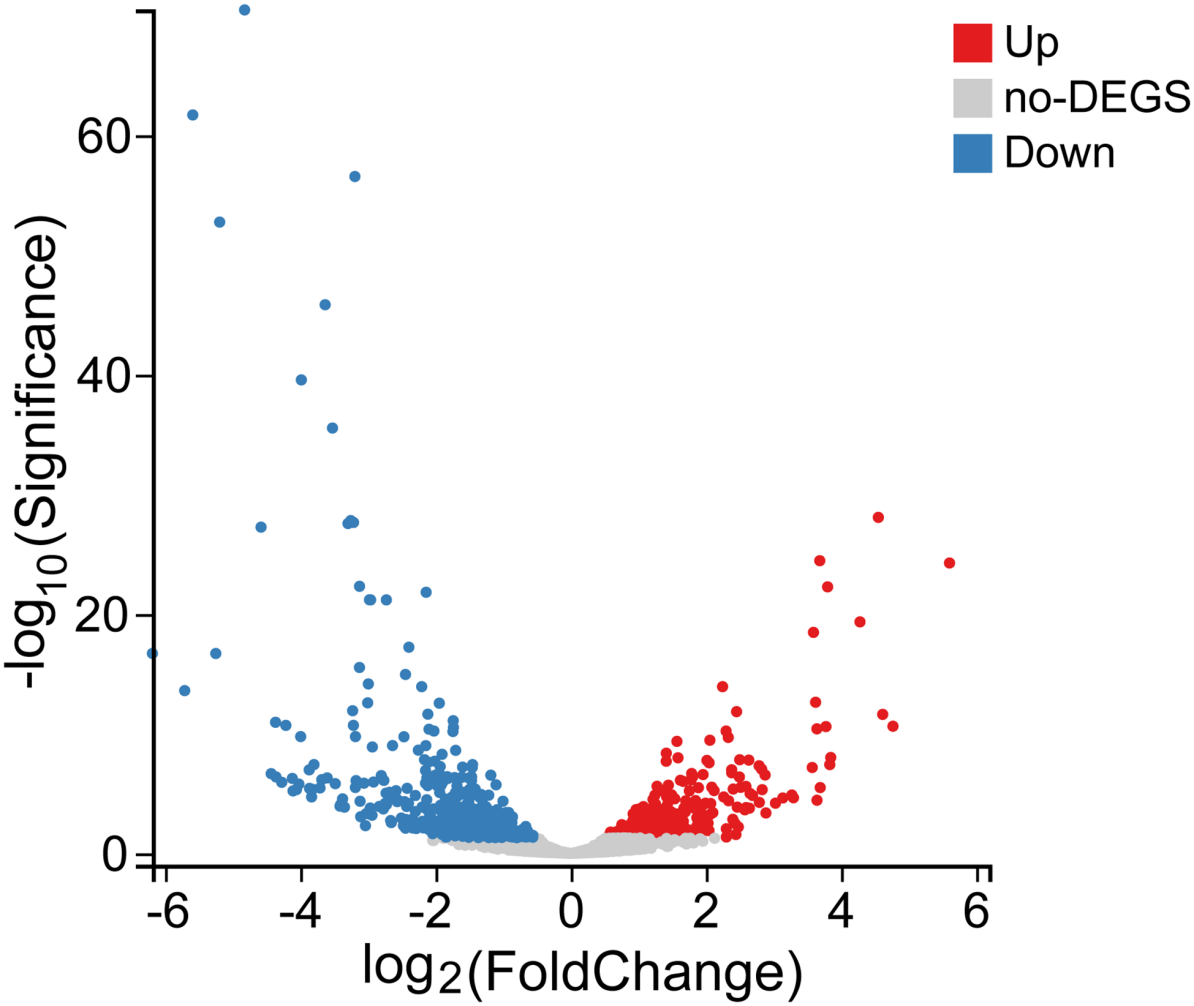
The volcano plots of genes for *Proteus* sp. YS02 between the control and Se treatment. Red and blue dots represent genes that were significantly upregulated and downregulated, respectively. Gray dots indicate the genes without significant differential expression.

**Figure 9:**
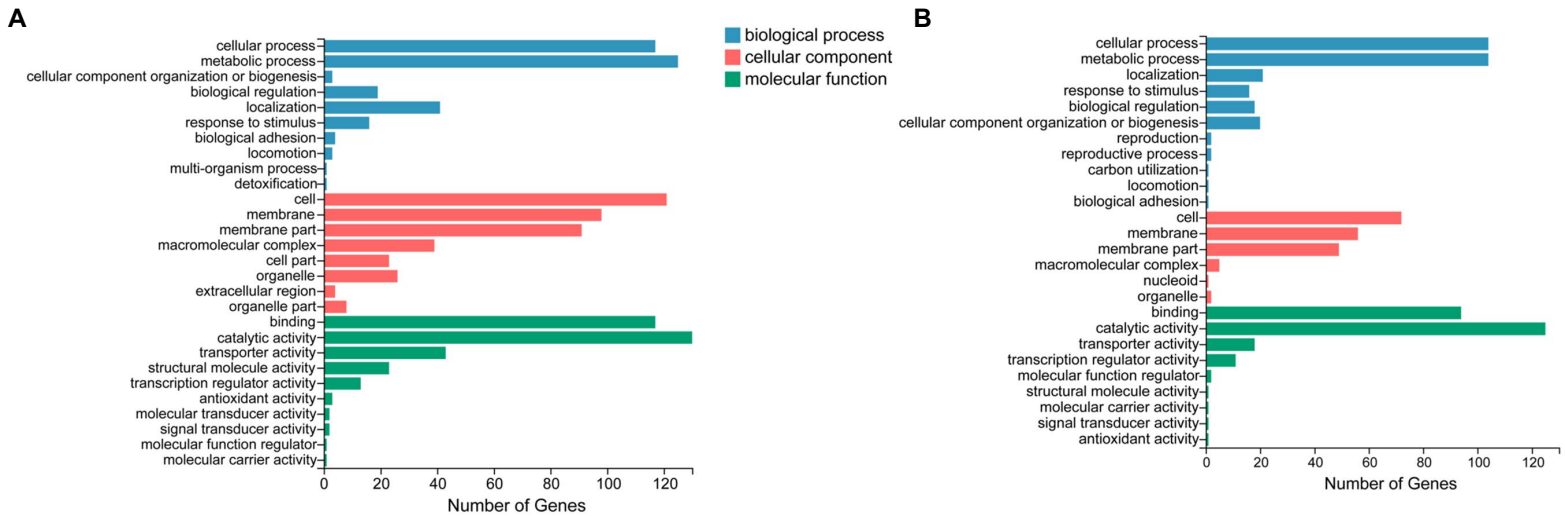
GO classifications of **(A)** downregulated DEGs and **(B)** upregulated DEGs. The X axis presents the number of DEGs belonging to specific categories. The Y axis presents three major functional categories of GO terms.

After assembling using Bowtie2, a total of 3,153 transcripts were screened and used for DEGs identification. Following comparison with CK (control sample without Se treatment), a total of 473 DEGs were found to have been expressed during Se treatment, among which 197 genes were significantly upregulated, and 276 genes were significantly downregulated (Figure 8). Based on GO functional analyses, these DEGs could be classified into three main GO categories, including molecular functions (MF), biological processes (BP), and cellular components (CC). For the 197 upregulated genes, DEGs enriched in BP mainly included cellular processes (52.8%), metabolic processes (52.8%), and localization (10.7%). CC DEGs were related to the cell (36.5%), membrane (28.4%), and membrane parts (24.9%), and MF DEGs were associated with catalytic activity (63.5%), binding (47.7%), and transporter activity (9.1%). The most obvious differences for the 276 downregulated genes of BP were those related to metabolic processes (45.3%), cellular processes (42.4%), and localization (14.9%); the most obvious differences of CC were also associated with the cell (43.8%), membrane (35.5%), membrane parts (33.0%), and macromolecular complexes (14.1%). The most obvious differences of MF were mainly concentrated in catalytic activity (47.1%), binding (42.4%), transporter activity (15.6%), and structural molecule activity (8.3%). GO enrichment of the top 20 GO terms showed that the functional proteins involved in the cellular response to stress (GO: 0033554), DNA metabolic process (GO: 0006259), ATPase-coupled sulfate transmembrane transporter (GO: 0015419), and ATP binding (GO: 0005524) were enhanced fundamentally in the presence of selenite, while genes assigned to macromolecular complexes (GO: 0032991), ribosomes (GO: 0005840), cytoplasmic parts (GO: 0044444), energy derivation (GO: 0015980), and the tricarboxylic acid cycle (GO: 0006099) were significantly downregulated (Figure 9).

### KEGG Pathway Enrichment Analysis

**Figure 10:**
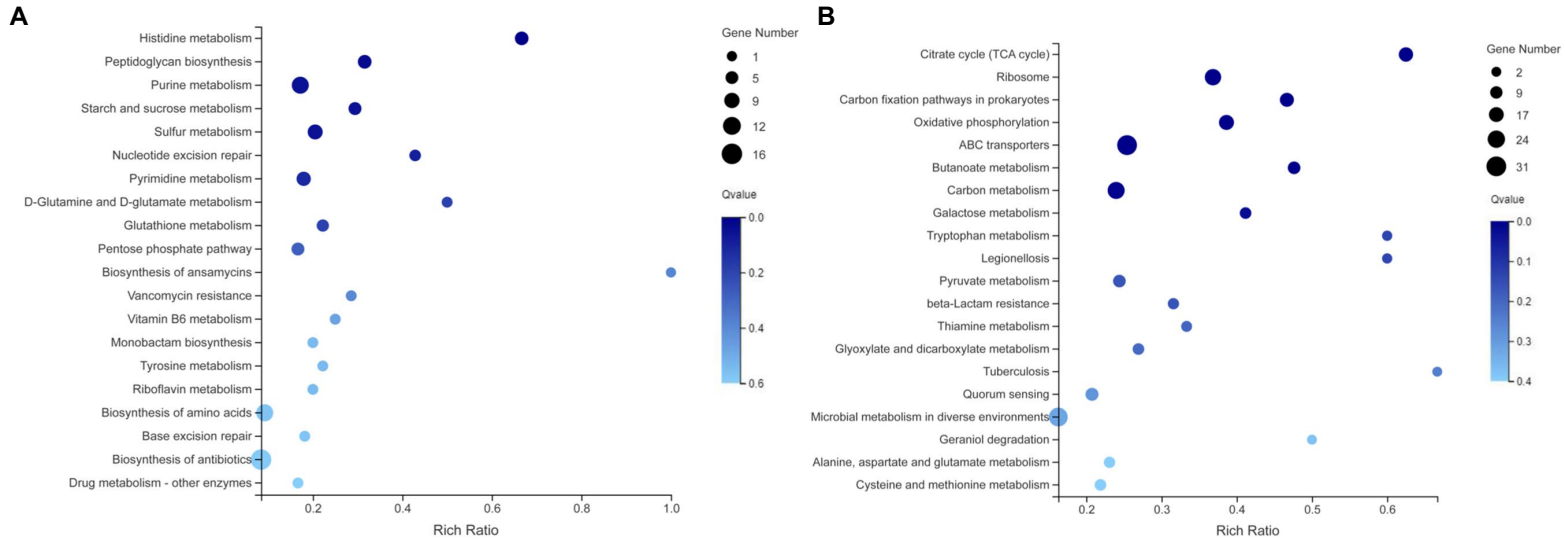
Enriched KEGG pathways for **(A)** upregulated DEGs and **(B)** downregulated DEGs in the presence of selenite. The X axis corresponds to the percentage of DEGs belonging to a specific pathway. The Y axis presents the names of the top 20 pathways.

KEGG pathway enrichment analysis for DEGs has shown that complicated metabolic pathways participated in the response to selenium stress. As shown in Figure 10A, the pathways that were significantly enriched among the upregulated DEGs included sulfur metabolism (ko00920, *p* < 0.05), purine metabolism (ko00230, *p* < 0.05), and histidine metabolism (ko00340, *p* < 0.05). The pathways involved in the biosynthesis of antibiotics (ko01130), pyrimidine metabolism (ko00240), and the PPP (ko00030) also had high enrichment scores but were not considered as significant. Furthermore, the enriched KEGG pathways for the downregulated DEGs between the control group and Se treatment group are shown in Figure 10B. ABC transporters (ko02010, *p* < 0.05), oxidative phosphorylation (ko00190, *p* < 0.05), carbon metabolism (ko01200, *p* < 0.05), and the citrate cycle (TCA cycle; ko00020, *p* < 0.05) were the most enriched pathways.

### The Main Genes Participating in Selenite Reduction and SeNPs Biosynthesis

Since selenium is one of the chalcogen elements, it is proposed that reductases in sulfur metabolic pathways may catalyze the reduction of Se(IV; Tan et al., 2018; Huang et al., 2021). In this study, the expressions of genes responsible for sulfate assimilation metabolism were significantly upregulated in the presence of selenite. The ABC transporter complex CysAWTP is composed of two CysA ATP-binding proteins, two transmembrane proteins (CysT and CysW), and a CysP solute-binding protein. It has been reported to be responsible for sulfate/thiosulfate import in many microbes (Han and Lee, 2006). It is interesting that selenite treatment upregulated expression of *cysA*, *cysW*, *cysU*, and *CysP* (the fold changes were 2.50, 2.79, 2.64, and 2.58 log_2_(FC) while comparing Se vs. CK, respectively), suggesting the sulfate transport system permease is responsible for the transmembrane transport of selenite in *Proteus* sp. YS02. The uptake and transport of selenite in *E. coli* have also been reported to be controlled by the sulfate transport complex ABC encoded by the *cysAWTP* operon (Tugarova and Kamnev, 2017). Furthermore, the gene expressions of *cysN* (encoding sulfate adenylyltransferase subunit 1), *cysD* (encoding sulfate adenylyltransferase subunit 2), *cysH* (encoding phosphoadenosine phosphosulfate reductase), *cysJ* [encoding sulfite reductase (NADPH) flavoprotein alpha-component], and *cysI* [encoding sulfite reductase (NADPH) hemoprotein beta-component], were all significantly upregulated (2.27, 2.65, 3.62, 3.59, and 3.69-fold, respectively). Sulfite reductase (cysJI in this case) was reported to catalyze the reduction of selenite/selenate to Se^0^ with NAD(P)H serving as an electron donor in bacterial isolates *Providencia rettgeri* HF16 (Huang et al., 2021) and *C. testosteroni* S44 (Tan et al., 2018). Similarly, in this study, the *CysJ* and *CysI* expression was significantly upregulated in the presence of selenite, suggesting that selenite is more likely to be transformed *via* a sulfite reductase-mediated metabolic pathway in YS02.

The PPP is one of the major sources of reducing power (NADPH) and metabolic intermediates that are required for biosynthetic processes (Kruger and von Schaewen, 2003; Wushensky et al., 2018). It is interesting that genes involved in the PPP pathway, such as *zwf* (encoding glucose-6-phosphate 1-dehydrogenase), *deoC* (encoding deoxyribose-phosphate aldolase), *tktA* (encoding transketolase), and deo*B* (encoding phosphopentomutase), were significantly upregulated. Furthermore, the expression of *aceE* (encoding pyruvate dehydrogenase E1 component) and *pdhC* (encoding pyruvate dehydrogenase E2 component) were also upregulated in the presence of selenite. The pyruvate dehydrogenase system catalyzes the oxidative decarboxylation of pyruvate with the production of acetyl coenzyme A (acetyl-CoA), NADH, and CO_2_ (Karsten et al., 2002). Taken together, the significant enhancement of the PPP genes and pyruvate dehydrogenase under selenite treatment confirmed their involvement in selenite biotransformation by supplying reducing equivalents and enhancing energy metabolism.

Additionally, the reduction and detoxification of selenite ions by microbes may be accompanied by the production of reactive oxygen species that can damage cell membranes or DNA (Tetteh et al., 2014; Zhao et al., 2018). It is noteworthy that the expression of several genes encoding enzymes classified as oxidoreductases and transferases, such as *nrd* (encoding ribonucleoside-diphosphate reductase), *hcp* (encoding hydroxylamine reductase), *cysG* (encoding uroporphyrin-III C-methyltransferase), *ndh* (encoding NADH dehydrogenase), and *hisG* (encoding ATP phosphoribosyltransferase) were also upregulated, indicating that these enzymes may be associated with oxidative stress defense and maintenance of redox homeostasis in strain YS02. However, the expression of several expected antioxidant protein-encoding genes, such as *gsh* (encoding glutathione synthetase), *gor* (encoding glutathione reductase), *gorA* (encoding glutathione-disulfide reductase), *sodB* (encoding superoxide dismutase), *trxA* (encoding thioredoxin), and *trxB* (encoding thioredoxin reductase), showed no significant change under selenite treatment. This was similar to the findings of Song et al. (2017b), who showed that selenite treatment caused a 2.42-fold increase in fumarate reductase abundance but had no effect on the expression of *gsh*, *gor*, or *trxB*.

### Validation of Candidate Genes Expression by qRT-PCR

The relative expressions of 10 selected DEGs were determined using RT-qPCR (Supplementary Table S2) to validate the RNA-seq sequencing results. The candidate DEGs included *cysN*，*CysA*，*CysP, CysI, CysJ*，*metF* (encoding methylenetetrahydrofolate reductase), *dppB* (encoding dipeptide transport system permease), *frdC* (encoding fumarate reductase subunit C), *frdD* (encoding fumarate reductase subunit D), and *sdhA* (encoding succinate dehydrogenase flavoprotein subunit). Overall, the upregulation or downregulation of the tested genes were consistent with the results obtained by transcriptome analysis, which indicated that the latter were reliable.

### Mechanism Dominating Selenite Reduction and SeNPs Production in *Proteus* sp. YS02

**Figure 11:**
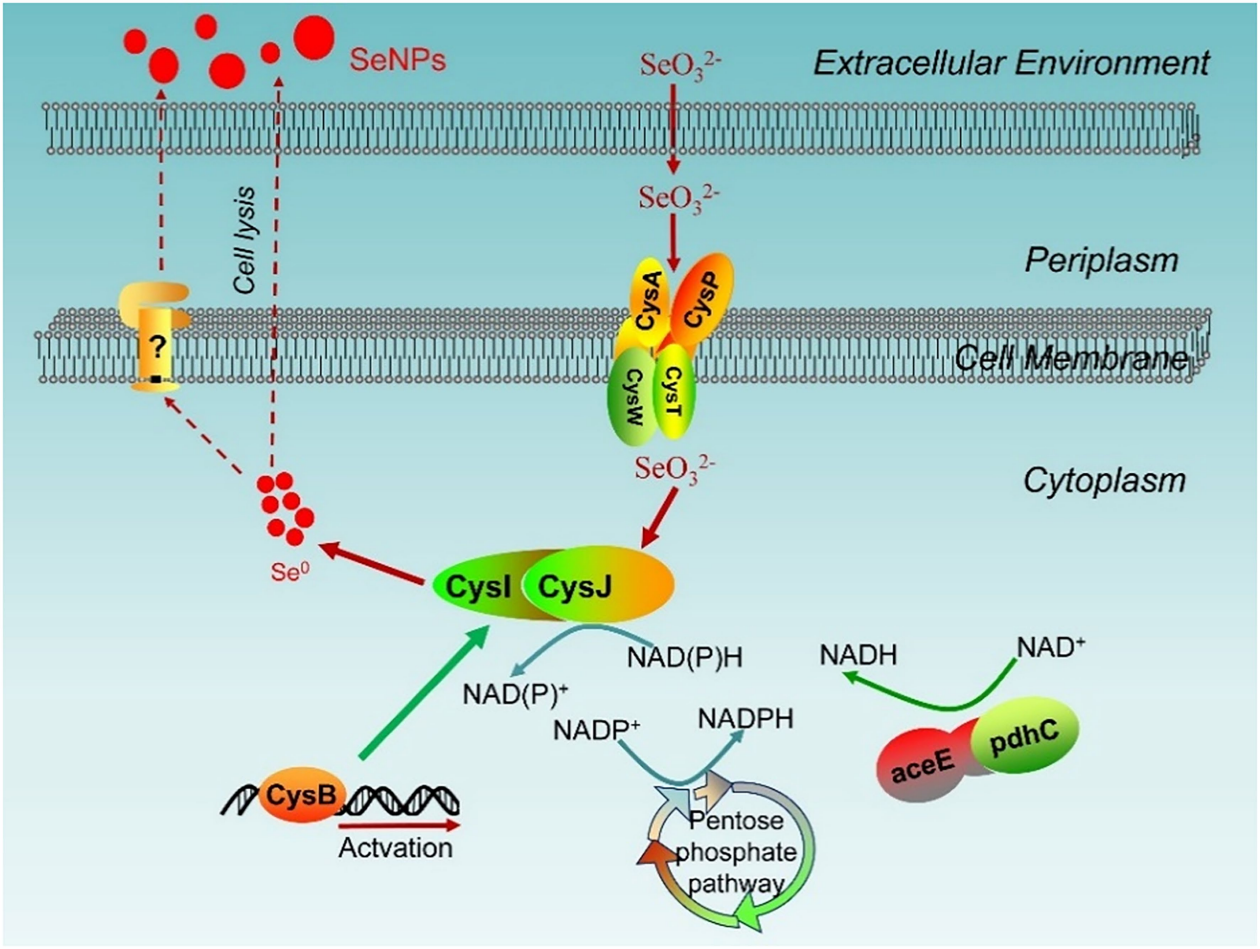
A hypothesized mechanism of selenite biotransformation and biosynthesis of SeNPs in strain *Proteus* sp. YS02. Yet unidentified processes are shown in dotted lines or question mark.

A model for selenite reduction and SeNPs production in *Proteus* sp. YS02 was proposed based on the above analysis. First, the sulfate transporter system (CysPUWA) is predicted to be involved in the uptake of Se(IV) from the extracellular fluid into the cytoplasm. Meanwhile, the PPP and pyruvate dehydrogenase were also activated under selenite treatment and thus produced NADPH or NADH, providing more reducing power for selenite reduction. The produced NADPH or NADH is then transported to sulfite reductase to favor the subsequent reaction. Finally, the reduction of Se(IV) to Se^0^ is accomplished by sulfite reductase (CysIJ in this case) with NADPH or NADH serving as the electron donor (Figure 11). Notably, TEM analysis detected Se^0^ nanoparticles both inside and outside of the cells and empty ghost cells, suggesting the produced SeNPs in the cytoplasm may be subsequently released into the extracellular space through cell lysis or by other vesicular secretion systems, as reported for other bacteria such as *V. natriegens* (Fernández-Llamosas et al., 2017). However, further research is needed to elucidate the mechanism responsible for the release of Se^0^ nanoparticles.

## Data Availability Statement

The datasets presented in this study can be found in online repositories. The names of the repository/repositories and accession number(s) can be found in the article/Supplementary Material.

## Author Contributions

YW and QY: conceptualization. YW: methodology and investigation. YS, YJ, and BM: software. JD and JC: data curation. AT and AK: FTIR spectroscopic analysis and interpretation. SH: writing–original draft preparation, supervision, and project administration. SH and AK: writing–review and editing. YW and SH: funding acquisition. All authors contributed to the article and approved the submitted version.

## Funding

This work was supported by the Anhui Provincial Natural Science Foundation, China (2108085QC88 and 2008085MC60), and the Key projects of Anhui Provincial Department of Education (KJ2021A0881). The work of AT and AK was carried out under research theme no. 121032300311-5 of the Russian Academy of Sciences. The funding organizations had no role in study design, data collection and analysis, decision to publish, or preparation of the manuscript.

## Conflict of Interest

The authors declare that the research was conducted in the absence of any commercial or financial relationships that could be construed as a potential conflict of interest.

## Publisher’s Note

All claims expressed in this article are solely those of the authors and do not necessarily represent those of their affiliated organizations, or those of the publisher, the editors and the reviewers. Any product that may be evaluated in this article, or claim that may be made by its manufacturer, is not guaranteed or endorsed by the publisher.

## Supplementary Material

The Supplementary Material for this article can be found online at:

https://www.frontiersin.org/articles/10.3389/fmicb.2022.845321/full#supplementary-material
