# Supplementary material for: Selenite Reduction by Proteus sp. YS02: New Insights Revealed by Comparative Transcriptomics and Antibacterial Effectiveness of the Biogenic Se0 Nanoparticles
Source: Front Microbiol. 2022 Mar 10;13:845321. doi: 10.3389/fmicb.2022.845321 (PMC8960269; doi:10.3389/fmicb.2022.845321)
Supplement: Supplementary file 1 [file Table_1.docx]

Table S1 The number of sequenced and mapped reads of genome-wide transcription analysis for each sample.

| Sample | Total Raw Reads (Mb) | Total Clean Reads (Mb) | Total Mapping (%) | Uniquely Mapping (%) | Clean Reads  Q20 (%) | Clean Reads  Q30 (%) |
| --- | --- | --- | --- | --- | --- | --- |
| CK-1 | 45 | 42.65 | 96.51 | 94.21 | 98.93 | 95.46 |
| CK-2 | 44.96 | 42.66 | 96.55 | 93.77 | 98.9 | 95.37 |
| CK-3 | 44.93 | 42.59 | 96.51 | 93.31 | 98.89 | 95.5 |
| Se-1 | 45.02 | 42.73 | 95.42 | 92 | 98.9 | 95.63 |
| Se-2 | 45.02 | 42.65 | 95.49 | 92.38 | 98.88 | 95.6 |
| Se-3 | 45.06 | 42.17 | 94.95 | 92.65 | 98.88 | 95.35 |
