# Supplementary material for: Selenite Reduction by Proteus sp. YS02: New Insights Revealed by Comparative Transcriptomics and Antibacterial Effectiveness of the Biogenic Se0 Nanoparticles
Source: Front Microbiol. 2022 Mar 10;13:845321. doi: 10.3389/fmicb.2022.845321 (PMC8960269; doi:10.3389/fmicb.2022.845321)
Supplement: Supplementary file 2 [file Table_2.docx]

| Gene ID | Description | Primer | Relative abundance log_2_ (FC) | |
| --- | --- | --- | --- | --- |
|  |  |  | RNA-Seq | qPCR |
| HMPREF0693_RS06520 | sulfate adenylyltransferase subunit 1 (*CysN*) | F: 5'-CTTACTATCGAATTTGTGCCTATC-3'  R:5'-AATGGTTGAACGCTGTCCTG-3' | 2.27 | 1.89 |
| HMPREF0693_RS04635 | sulfate transport system ATP-binding protein (*CysA*) | F: 5'-CATTTAGCACAGCGTTATCC-3'  R: 5'-CAGTTCTCACTTTGGCATCT-3' | 2.50 | 2.29 |
| HMPREF0693_RS04650 | sulfate transport system substrate-binding protein (*CysP*) | F: 5'-ATTATCAAGTTATTGTCCCTCC-3'  R:5'-TTATCTAGCGTGCCATCTGT-3' | 2.58 | 2.11 |
| HMPREF0693_RS06540 | sulfite reductase (NADPH) hemoprotein beta-component (*CysI*) | F: 5'-AGAGGCACAGAAAGAGCAGA-3'  R:5'-CGACCACAACCGTTAGGACA-3' | 3.69 | 3.44 |
| HMPREF0693_RS06545 | sulfite reductase (NADPH) flavoprotein alpha-component (*CysJ*) | F: 5'-CGTCTTTACTCCATCGCTTCA-3'  R:5'-CGTCGCACCTTCACTTTCAC-3' | 3.59 | 3.66 |
| HMPREF0693_RS16465 | methylenetetrahydrofolate reductase (*metF*) | F: 5'-TTTGTTTCGGTCACTTATGG-3'  R:5'-ATCTGGCTTCTGGCTGTTAT-3'' | -6.19 | -5.28 |
| HMPREF0693_RS16885 | dipeptide transport system permease (*dppB*) | F: 5'-GGATATTCTATGCCTATCTTCTG-3'  R:5'-TTCTCCCAATACTTCCAACA-3' | -3.63 | -3.25 |
| HMPREF0693_RS10865 | Fumarate reductase subunit C (*frdC*) | F: 5'-CAAACTGGTGGACGAAACTC-3'  R:5'-TTGCGATAAGGGTCACAATA-3' | -1.94 | -2.15 |
| HMPREF0693_RS10870 | Fumarate reductase subunit D (*frdD*) | F: 5'-ATGTGGAGTGCGATTGTCTC-3'  R:5'-TGTATGGTGAATACGGTGTAA-3' | -.2.11 | -1.79 |
| HMPREF0693_RS06715 | Succinate dehydrogenase flavoprotein subunit (*sdhA*) | F: 5'-GTTAGTGACGGAAGGTTGCC-3'  R:5'-GTGGTCGAGTTTCAGTTTGG-3' | -2.42 | -2.21 |
|  | 16S rRNA | F: 5′-AGAGTTTGATCCTGGCTCAG-3′  R: 5′-CTGCTGCCTCCCGTAGGAGT-3′ |  |  |

Table S2 Differentially expressed genes in YS02 in the presence of selenite determined by RNA-seq and q-PCR analysis.
